# Whole Grain Intake and Impaired Fasting Glucose in Adolescents, National Health and Nutrition Examination Survey, 2005–2014

**DOI:** 10.5888/pcd17.190439

**Published:** 2020-10-22

**Authors:** June M. Tester, Katharine B. Stiers, Andrea Garber, Cindy W. Leung

**Affiliations:** 1 UCSF Benioff Children’s Hospital Oakland, Oakland, California; 2 Keck School of Medicine, University of Southern California, Los Angeles, California; 3 Department of Pediatrics, University of California San Francisco, San Francisco, California; 4 Department of Nutritional Sciences, School of Public Health, University of Michigan, Ann Arbor, Michigan

## Abstract

**Introduction:**

Large prospective cohort studies show a lower risk of developing type 2 diabetes among adults with higher whole grain consumption. Less is known about the relationship between whole grain consumption and precursors for diabetes risk in adolescents. We examined whether intake of whole grains was associated with impaired fasting glucose (IFG) in adolescents.

**Methods:**

We analyzed data on dietary intake from an average of two 24-hour diet recalls from fasting, nondiabetic adolescents aged 12–18 years (N = 2,286) across 5 cycles of the National Health and Nutrition Examination Survey (NHANES 2005–2014). We used logistic regression to calculate the odds of having IFG (100–125 mg/dL) with respect to servings of whole and refined grains, as well as percentage of whole grains, adjusting for sex, age, race/ethnicity, annual household income, obesity, total energy, and diet quality.

**Results:**

IFG was present in 17% of participants. After adjusting for covariates, number of servings per day of whole grains was significantly associated with lower odds of IFG, but there was no relationship between IFG and servings of refined grains or percentage of whole grains. Consuming at least 1 ounce-equivalent serving (16 g) of whole grains daily, compared with consuming no whole grains, was associated with a 40% reduction in the adjusted odds of having IFG (adjusted odds ratio = 0.60; 95% CI, 0.38–0.93).

**Conclusion:**

Analysis of 10 years of national cross-sectional data suggests that US adolescents whose daily diets consist of a minimum threshold amount of whole grains may be less likely to have IFG, a finding that has implications for diabetes prevention in adolescents.

**Summary**

**What is known on this topic?**

Evidence from years of follow-up shows that adults who consume more whole grains have a lower risk of developing type 2 diabetes. However, the relationship between grain consumption and prediabetes in adolescents is unknown.

**What is added by this report?**

We evaluated data from a nationally representative sample and found that whole grain intake is associated with lower odds of having impaired fasting glucose in adolescents.

**What are the implications for public health practice?**

It may be beneficial for adolescents to consume at least 1 serving per day of whole grains.

## Introduction

Observational and experimental data suggest a protective relationship between whole grain consumption and type 2 diabetes. Decades of prospective cohort data with the Nurses’ Health Study (with more than 1 million person years of follow-up) demonstrated a 28% lower relative risk of type 2 diabetes incidence among women who consume more whole grains, even after adjusting for weight status, which is a strong predisposing factor for development of diabetes (1). Among men in the Health Professionals Follow-up Study, consumption of whole grains exerted a stronger protective effect against incident diabetes than other components of the examined “prudent dietary pattern” (ie, fruit, vegetables, and fish) (2). Recent evidence from European cohort data suggests that consuming 3 servings per day of whole grain foods (compared with consuming a half serving) could reduce risk of incident type 2 diabetes by 20% (3). Evidence about whole grain cereal fibers in particular shows a high protective effect. A recently published meta-analysis of 8 cohort studies with an average of 12.6 years of follow-up (adults aged 35–79 years) demonstrated a 32% lower risk of developing type 2 diabetes (4) that was nearly identical to the estimate from a meta-analysis published more than a decade ago (5).

However, considerably less is known about the relationship between whole grain consumption and risk for type 2 diabetes in children and adolescents. This discrepancy represents a gap in knowledge and a critical opportunity for more investigation, given the rising prevalence of type 2 diabetes among US youth (6). To our knowledge, the only study that has examined whole grain intake and diabetes risk in youth examined 285 Minnesota adolescents and demonstrated that whole grain intake was associated with greater insulin sensitivity (measured with euglycemic clamp). This relationship was strongest among adolescents with high body mass index (BMI, kg/m^2^), although it was still significant even after adjustment for adiposity, which independently increases diabetes risk (7). Although type 2 diabetes is rising in adolescents, it is still a rare outcome (estimated in 2012 to be 12.5 cases per 100,000 youth) (6). However, although insulin sensitivity is a relevant marker for predicting type 2 diabetes, not all clinically meaningful diagnostic end points have been considered. Fasting glucose is collected widely in large population samples such as the National Health and Nutrition Examination Survey (NHANES), and having a fasting glucose between 100 and 125 mg/dL meets diagnostic criteria for prediabetes (8).

Despite the demonstrated health benefits associated with habitual whole grain consumption, daily intake in the United States is inadequate and estimated to be approximately 1 ounce-equivalent serving (1 oz-eq) per day for adults (9) and as low as a half serving (0.5 oz-eq) per day for adolescents (10). An ounce-equivalent serving of whole grains contains at least 16 g of whole grains (11). Studies in adults suggest that there is lower risk of developing type 2 diabetes when consumption of whole grains is 2 to 3 servings per day (5,12). Evidence from the aforementioned experimental study of adolescents demonstrated that consumption of 1.5 oz-eq per day of whole grains was associated with lower BMI and greater insulin sensitivity (7), but further exploration using diagnostic clinical end points is warranted to more fully understand the diabetes risk spectrum.

This analysis was conducted to address 2 gaps: 1) characterizing the unexplored relationship between whole grain intake and a clinical marker of diabetes risk in adolescents, and 2) evaluating a potential threshold at which a minimum level of intake confers benefit. This study aimed to test our central hypothesis that whole grain intake would be positively associated with decreased odds of impaired fasting glucose (IFG) among adolescents.

## Methods

### Study population

We examined data on adolescents aged 12 to 18 years who participated in NHANES, a complex, multistage probability, cross-sectional survey designed to be representative of the US civilian, noninstitutionalized population. We examined five 2-year data cycles (2005–2006, 2007–2008, 2009–2010, 2011–2012, and 2013–2014) and limited the sample to participants who were selected to be in the fasting subsample (morning visit) and had fasted for at least 8 hours. Pubertal status was added to the continuous NHANES questionnaire only starting in 2011–2012, with provided Tanner staging drawings, and data have not yet been released to the public. 

Individuals were excluded if they had a self-reported or laboratory-confirmed (glucose ≥126 mg/dL) diagnosis of diabetes (n = 14); were pregnant (n = 12); or were missing data on height and/or weight (n = 27), complete diet recall (n = 87), income (n = 152), or laboratory data (n = 5). This yielded a final sample of 2,286 participants. 

### Measures

Demographic information obtained during the survey included the adolescent’s age and sex. Race/ethnicity were used to categorize participants into 4 categories: Hispanic, non-Hispanic White, Non-Hispanic Black, and other. Annual household income and family size are used with each cycle of NHANES to calculate the index of family income to the federal poverty level (FPL) in accordance with the poverty guidelines of the US Department of Health and Human Services (13).

Height and weight were measured by trained personnel at the mobile examination center using standardized protocols (14). Age- and sex-specific BMI percentiles were calculated using codes provided by the Centers for Disease Control and Prevention, classifying participants as normal weight (BMI <85th percentile), overweight (BMI 85th to <95th percentile), obesity (BMI ≥95th percentile to <120% of the 95th percentile), and severe obesity (BMI ≥120% of the 95th percentile) (15).

All participants included in the subsample had been fasting for at least 8 hours (16). Plasma fasting glucose was examined using hexokinase-mediated reaction, which is the reference analytic method. Per recommended analytic guidelines, we used Deming regression equations to be able to include fasting glucose data from 2005–2006 with data from 2007–2008 (17). According to the American Diabetes Association definition, fasting glucose between 100 mg/dL and 125 mg/dL is considered impaired and meets the criteria for prediabetes (8).

A 24-hour diet recall was conducted in person at the mobile examination center, and a second recall was collected via telephone. The Food Pattern Equivalents Database (FPED) was used to convert foods and beverages reported in NHANES to US Department of Agriculture food pattern components (18). The FPED database reports grain consumption in terms of ounce-equivalents to align with the food group serving definitions used in the 2005 Dietary Guidelines for Americans (eg, one slice of bread; a cup of cereal or a half cup of hot cereal, cooked pasta, rice, or other grain such as bulgur, oatmeal, and cornmeal) (18). The average of the 2 recalls was used for this analysis.

The primary dietary outcome of interest was consumption of whole grains, reported in the FPED database as ounce-equivalents of whole grains. Whole grains were also examined as a proportion of total grain intake, calculated as the ratio of ounce-equivalents of whole grains divided by the sum of whole grains and non-whole (refined) grains. The mean intake of refined grains (oz-eq/d) was also examined. The Healthy Eating Index 2010 (HEI 2010), which is scored from 0 to 100 points and quantifies adherence to the 2010 Dietary Guidelines for Americans, was calculated using a publicly available scoring algorithm (19). 

We also tested to determine a meaningful threshold of whole grain consumption. We used 0 oz-eq (<31st percentile), 0.5 oz-eq (57th percentile), and 1.0 oz-eq (77th percentile) as cutoff points to approximate the 25th, 50th, and 75th percentiles in the following 4 categories: 0 oz-eq/d, 0 to less than 0.5 oz-eq/d, 0.5 oz-eq/d to less than 1 oz-eq/d, and 1.0 oz-eq/d or more.

### Statistical analyses

We used survey weights to account for the complex, multistage probability sampling design used in NHANES in accordance with recommendations from the National Center for Health Statistics (16). We used fasting subsample exam weights to appropriately reflect sampling methodology. Analyses were conducted using Stata 15.1 (StataCorp LLC).

For descriptive analyses, participants (all non-diabetic) with normal fasting glucose were compared with those with IFG by using design-based *χ* ^2^ tests for categorical variables (eg, race/ethnicity) and survey-weighted univariate linear regression for continuous variables (eg, age).

Logistic regression was conducted to evaluate odds of prediabetes (defined here by IFG) associated with the following continuous variables: whole grain consumption, refined grain consumption, and percentage of total grains that were whole. Multivariable models were adjusted for sex, age, race/ethnicity, income, and BMI, as these are known to be associated with prediabetes and whole grain intake (20–22). Furthermore, models were adjusted for total energy intake (average kcals consumed per day) and overall dietary quality (using HEI 2010), as is commonly done in nutritional epidemiologic studies (23). Because pubertal status was not available and because the pubertal growth spurt is known to decrease insulin sensitivity (24), sensitivity analysis was conducted to evaluate the subset of participants who were aged 16 to 18 years and presumably all post-pubertal. We assessed obesity as a potential mediator between whole grain consumption and IFG, using the causal mediation approach (medeff with jackknife) by Hicks and Tingley (25). Significance for all results was set at *P* < .05.

## Results

### Demographics

Participants included in the final sample (N = 2,286) and the 297 excluded individuals differed by race/ethnicity (*P* = .01): those who were excluded had a smaller percentage of non-Hispanic White individuals (47% compared with 60%) and a larger percentage of Hispanic (25% vs 19%), non-Hispanic Black (17% vs 14%), and other/mixed (10% vs 7%) individuals. However, on every other characteristic, as well as the proportion with IFG, there were no significant differences between those included and those excluded.

The survey-weighted prevalence of IFG in the sample was 17.2%. The presence of impaired compared with normal fasting glucose varied significantly (*χ* ^2^ test, *P* < .05) by sex (ie, 71% of those with IFG were male), by race/ethnicity (ie, Hispanics comprised 16.9% of those with normal glucose but 26.9% of those with IFG), and degree of obesity (ie, the proportion with IFG compared with normal glucose was double among those with severe obesity) (Table 1). Individuals with IFG also had significantly higher mean daily caloric intake (2,098 kcal vs 1,974 kcal; *P* < .05), and lower income level (231% of the FPL vs 266% of the FPL).

**Table 1:** Characteristics of Adolescents Aged 12–18 Years, by Impaired Fasting Glucose Level, National Health and Nutrition Examination Survey, 2005–2014^a^

Characteristic	Total Sample, N = 2,286	Normal Fasting Glucose, n = 1,886 (83%)	Impaired Fasting Glucose, n = 400 (17%)	*P* Value
**Demographics and Health**				
**Sex, n (%)**				
Male	1,187 (51.8)	910 (47.8)	277 (70.7)	<.001
Female	1,099 (48.2)	976 (52.2)	123 (29.3)	
**Race/ethnicity, n (%)**				
White	660 (60.1)	551 (61.0)	109 (55.8)	<.001
Hispanic	802 (18.6)	625 (16.9)	177 (26.9)	
Non-Hispanic Black	620 (14.4)	545 (15.3)	75 (10.0)	
Other/mixed	204 (6.9)	165 (7.0)	39 (7.3)	
**Weight status,^b^ n (%)**				
Normal	1,428 (65.4)	1,214 (67.8)	214 (54.4)	<.001
Overweight	386 (15.5)	325 (15.5)	61 (15.5)	
Obesity	287 (11.9)	212 (10.5)	75 (18.3)	
Severe obesity	185 (7.2)	135 (6.2)	50 (11.8)	
**Age, y, mean (SE)**	15.0 (0.06)	15.1 (0.06)	14.8 (0.13)	.07
**Income level, % FPL, mean (SE)**	260 (6.7)	266 (7.3)	231 (14.2)	.03
**Diet Quality/Dietary Intake**				
**HEI 2010, mean (SE)**	45.1 (0.4)	45.3 (0.45)	44.2 (0.74)	.19
**Energy intake, kcal/d, mean (SE)**	1,995 (24)	1,974 (25)	2,098 (58)	.03
**Whole grain intake**				
Whole grains, oz-eq/d, mean (SE)	0.67 (0.04)	0.69 (0.04)	0.54 (0.05)	.01
Refined grains, oz-eq/d, mean (SE)	6.40 (0.1)	6.32 (0.10)	6.93 (0.26)	.03
Percentage whole grains, mean (SE)	9.2 (0.5)	9.1 (0.58)	9.5 (1.5)	.83

Abbreviations: FPL, federal poverty level; HEI, Healthy Eating Index; oz-eq, ounce-equivalent; SE, standard error.

a All percentages are weighted. *P* values determined by using *χ* ^2^ test.

b Obesity defined as a body mass index ≥95th percentile to <120% of the 95th percentile. Severe obesity defined as a body mass index ≥120% of the 95th percentile.

Whole and refined grain consumption also varied with respect to variables associated with IFG. Consumption of whole grains was associated with lower BMI percentile, higher income, higher diet quality (as measured by HEI 2010), and higher total energy intake (*P* < .05 for all). Compared with non-Hispanic Whites, Hispanics and non-Hispanic Blacks had lower intake of whole grains (*P* < .05). Consumption of refined grains was associated with lower diet quality (per HEI 2010) and higher total energy intake (ie, calories) (*P* < .05), but it was not associated with income or BMI. Compared with non-Hispanic Whites, Hispanics had higher intake of refined grains (*P* < .05), but they were the only group with this association.

### Whole and refined grains and odds of IFG

Individuals with IFG had lower whole grain intake (0.54 vs 0.69 oz-eq/d) and higher refined grain intake (6.93 vs 6.32 oz-eq/d) (*P* < .05 for both) (Table 1). In unadjusted logistic regression, each incremental serving of whole grains was associated with a 19% reduction in the odds of IFG (odds ratio [OR] = 0.81; 95% CI, 0.69–0.96). This finding remained significant in a multivariable model that adjusted for sex, age, race/ethnicity, obesity category, total energy (kcal/d), and dietary quality index (per HEI 2010), with a 23% reduction in odds of IFG (aOR = 0.77; 95% CI, 0.65–0.91) (Table 2). The adjusted odds of IFG among adolescents aged 16 to 18 years was of comparable magnitude and was also significant (aOR = 0.72; 95% CI, 0.54–0.96).

**Table 2:** Odds of Impaired Fasting Glucose Associated With Increase in Grain Intake Among Adolescents Aged 12–18 Years (N = 2,286), National Health and Nutrition Examination Survey, 2005–2014

Type of Grain^a^	Unadjusted OR (95% CI)	*P* Value	Adjusted OR^b^ (95% CI)	*P* Value
Whole grains, 1 ounce-equivalent per day increase	0.81 (0.69–0.96)	.02	0.77 (0.65–0.91)	.002
Refined grains, 1 ounce-equivalent per day increase	1.04 (1.01–1.08)	.02	1.02 (0.96–1.08)	.54
Percentage whole grains, 10% increase	1.02 (0.89–1.16)	.82	1.04 (0.91–1.20)	.56

Abbreviation: OR, odds ratio.

a One ounce-equivalent = 16 grams.

b Adjusted for sex, age, race/ethnicity, income, obesity category, total energy (kcal/d), and dietary quality index (Healthy Eating Index 2010).

Unadjusted analysis indicated a small increase (4%) in odds of IFG with each additional serving of refined grains (OR = 1.04; 95% CI, 1.01–1.08), but the effect was no longer significant in the adjusted multivariable model (aOR = 1.02; 95% CI, 0.96–1.08) (Table 2). Percentage of whole grains was not significantly associated with odds of IFG in either unadjusted or adjusted models (Table 2).

Whole grains were associated with both obesity and IFG. The interaction between whole grain consumption and obesity (exposure–mediator) was small and not significant, and the percentage of the total effect of whole grain consumption mediated by obesity was less than 1%. Refined grains were not significantly associated with obesity in either univariate or adjusted analyses.

### Examining for threshold effect with whole grain intake

Odds of IFG by percentile of whole grain consumption (25th, 50th, and 75th) are presented in Table 3. Compared with participants who had consumed no whole grains, participants whose whole grain consumption was less than 1 oz-eq/d did not appear to have any protective effect from their whole grain consumption. However, compared with peers with no whole grain consumption, adolescents who had consumed at least 1 oz-eq/d had a 40% reduction in odds of having IFG (aOR = 0.60; 95% CI, 0.38–0.93), even after adjusting for sex, race/ethnicity, diet (energy intake and diet quality index), and adiposity. Sensitivity analysis of the subsample of older adolescents aged 16 to 18 years also showed significant reduction in adjusted odds of IFG (aOR = 0.49; 95% CI, 0.24–0.97).

**Table 3:** Odds of Impaired Fasting Glucose Among Adolescents Aged 12–18 Years (N = 2,286), by Threshold of Daily Whole Grain Intake, National Health and Nutrition Examination Survey, 2005–2014^a^

Whole Grain Intake	Adjusted Odds Ratio^b^ (95% CI)	*P* Value
0 ounce-equivalents per day	1 [Reference]	NA
0 to <0.5 ounce-equivalents per day	1.07 (0.70–1.63)	.74
0.5 to <1.0 ounce-equivalents per day	1.21 (0.77–1.90)	.40
≥1.0 ounce-equivalents per day	0.60 (0.38–0.93)	.02

Abbreviation: NA, not applicable.

a Thresholds of grain intake correspond approximately to the 25th, 50th, and 75th percentiles; 1 ounce-equivalent = 16 grams.

b Adjusted for sex, age, race/ethnicity, income, obesity category, total energy (kcal/d), and dietary quality index (Healthy Eating Index 2010).

## Discussion

Results from this study demonstrate a significant association between whole grain consumption and IFG among US adolescents, and the study was the first to examine a clinical end point, IFG, in a nationally representative sample of adolescents. The prevalence of type 2 diabetes among children and adolescents in the United States is rising (6), and as many as one-quarter of adolescents have prediabetes (26). This analysis highlights the need for consideration of whole grain consumption in education and intervention efforts regarding diabetes prevention in youth.

We aimed to determine whether a threshold of consumption exists above which adolescents experience meaningful benefit; we found lower odds of having IFG among adolescents who consume at least 1 oz-eq serving of whole grains per day. In 2000, around the time of the introduction of whole grains as a priority area in the Dietary Guidelines for Americans (27), whole grain consumption in the United States was approximately one-half serving per day for children aged 6 to 18 (10). Modest gains in consumption have occurred over time, with levels closer to three-quarters of a serving per day for children in 2011–2012 (10). However, socioeconomic disparities with respect to whole grain consumption are growing. An analysis of whole grain consumption in US adolescents from 2005–2012 showed that, although whole grain consumption rose during this interval, low-income (<200% of the FPL) adolescents continued to eat only 0.5 servings per day while their more affluent peers had doubled intake to 1.0 servings per day (22). This discrepancy highlights a priority for strategies to increase whole grain consumption among low-income adolescents to reduce future disparities in diabetes risks by socioeconomic status.

Many proposed mechanisms explain why whole grains may be beneficial for glucose control. Researchers have long recognized that fiber-rich whole grains increase satiety and fullness, are high in antioxidants, and have dietary fibers that are fermented in the digestive tract, leading to a slower and steadier rise in blood glucose compared with the rise from processed grains with a higher glycemic index (ie, ranking of carbohydrate-containing foods according to their effect on blood glucose levels). Whole grains are also high in bioactive compounds like magnesium (which is particularly high in whole grain cereals), which increases insulin secretion and glucose clearance from the blood (28). Newer hypotheses are focusing on examining how the many bioactive compounds found in whole grains may operate synergistically as a “whole grain package” (29).

Excess adiposity is a risk factor for diabetes, with improvement in risk seen with relatively small (5%–7%) decreases in weight (30). In this sample, odds of IFG increased proportionally with weight status, and adolescents with severe obesity had 2.5 times the odds of having IFG compared with their normal-weight counterparts. The inverse relationship between (higher) whole grain consumption and (lower) odds of IFG remained significant after adjustment for weight status (and for dietary quality and caloric intake). Furthermore, mediation analysis showed that, although whole grains were associated with both obesity and IFG, obesity was not mediating the relationship between whole grain consumption and IFG. This finding suggests that beyond the known benefits a diet high in whole grains can have with respect to weight status, there may be other independent benefits that consumption of whole grains has with respect to glucose regulation in adolescents.

Practitioners and clinicians should be mindful of the impact of their recommendations to patients regarding whole grain consumption. A simple message to merely add whole grain foods to an unhealthy baseline of refined grains increases the total amount of grain-based carbohydrates without replacing refined grains with whole grains and, thus, misses the mark. Further complicating the task of messaging is the lack of consistency in what makes it possible to market a food as being “whole grain” (31). Foods containing exclusively whole grains (ie, 100% whole grain) have the highest nutritional value and are preferentially recommended by the Dietary Guidelines for Americans (11). However, package labeling regulations with minimal requirements regarding whole grain content (eg, promotion of products with as little as one-half serving of whole grains) frequently draw consumer attention to processed foods containing some whole grains but which are higher in sugar and calories (32).

This analysis has limitations and strengths. First, the data are cross-sectional, so causal relationships cannot be determined. Higher whole grain consumption in adolescents appears to be associated with lower odds of having IFG, but we cannot conclude from these data that increasing one’s whole grain intake will directly lower the risk of developing IFG. To determine this, controlled experimental studies are required. Another limitation is that physical activity was not included as a covariate because the question items regarding moderate to vigorous physical activity (and screen time) changed, so there was no consistent variable during the time frame studied. Obesity was assessed with BMI, which does not provide differentiation between fat mass and lean mass, another limitation of the study. Strengths of this analysis are the complex sampling technique of NHANES, which allows for creating generalizable, nationally representative estimates. Although many large studies estimate whole grain intake with diet screeners that may include only a few whole grain foods such as cereal fibers, these data include quantification of whole grain intake with two 24-hour diet recalls.

Our analysis of a nationally representative sample of adolescents demonstrated that higher whole grain consumption is associated with lower odds of having IFG, which constitutes a clinical diagnosis of prediabetes, even after adjusting for obesity. Specifically, eating 1 serving of whole grains per day may reduce the odds of IFG by 40%. These findings suggest that a diet high in whole grains may be associated with lower diabetes risk in American adolescents.
